# “Helping my neighbour is like giving a loan*…*” –the role of social relations in chronic illness in rural Uganda

**DOI:** 10.1186/s12913-017-2666-5

**Published:** 2017-11-09

**Authors:** Jovita Amurwon, Flora Hajdu, Dominic Bukenya Yiga, Janet Seeley

**Affiliations:** 1 Urban and Rural Development Unit, Swedish University of Agricultural Sciences, Uppsala, Sweden; 2 Centre for International Health, University of Bergen, Bergen, Norway; 3 Medical Research Council/Uganda Virus Research Institute, Entebbe, Uganda; 4 London School of Hygiene and Tropical Medicine, Bloomsbury, UK

**Keywords:** Uganda, Chronic illness, Access to care, Social relations, Health systems

## Abstract

**Background:**

Understanding individuals’ experience of accessing care and tending to various other needs during chronic illness in a rural context is important for health systems aiming to increase access to healthcare and protect poor populations from unreasonable financial hardship. This study explored the impact on households of access to free healthcare and how they managed to meet needs during chronic illness.

**Methods:**

Rich data from the life stories of individuals from 22 households in rural south-western Uganda collected in 2009 were analysed.

**Results:**

The data revealed that individuals and households depend heavily on their social relations in order to meet their needs during illness, including accessing the free healthcare and maintaining vital livelihood activities. The life stories illustrated ways in which households draw upon social relations to achieve the broader social protection necessary to prevent expenses becoming catastrophic, but also demonstrated the uncertainty in relying solely on informal relations.

**Conclusion:**

Improving access to healthcare in a rural context greatly depends on broader social protection. Thus, the informal social protection that already exists in the form of strong reciprocal social relations must be acknowledged, supported and included in health policy planning.

## Background

Low-income countries such as Uganda, along with other sub-Saharan African countries, are experiencing an increase in chronic illnesses [1]. Chronic illness can be defined as conditions that require ongoing and long-term healthcare management [2]. As diseases such as human immunodeficiency virus (HIV) become chronic conditions, and with the increasing prevalence of non-communicable diseases (NCDs), the demand for healthcare resources is increasing. Non-communicable diseases caused 38 million deaths globally in 2012, of which 80% were in low-income countries [3]. In 2010, NCDs killed about one million people in Uganda [4]. Chronic illness often requires sustained expenditure on healthcare, and due to little financial risk protection in low-income countries, this financial burden falls on individuals, resulting in a decline in economic outcome and in resilience for affected households [5, 6].

Healthcare systems around the world are seeking to ensure that everyone has access to the health services they need without experiencing unreasonable financial hardship, and providing free treatment and medication at health facilities is one of the strategies used in countries like Uganda [7, 8]. However, population groups such as those in rural areas, non-medical factors such as transportation comprise the largest financial barrier to accessing healthcare. Improving access to healthcare in such population groups requires wider social protection. There are various strategies and actions that enhance the capacity of poor and vulnerable groups to better manage risks and shocks. For example, rural populations are known to employ a strategy of seeking resources from their social relations in order to address their needs during chronic illness [9–12].

There is limited knowledge on the extent to, and the mechanisms through which social relations minimise barriers to healthcare access and provide financial protection for rural populations in a low-income setting [13]. In this article, we aim to establish the role of social relations in providing such wider social protection, as well as the importance of availing free healthcare in a rural population. This is done through examining how households support themselves and meet their various needs during chronic illness. We use rich data from the life stories of individuals and their households within a surveillance cohort accessing free treatment and medication in south-western Uganda. Such studies are useful considering the growing interest in health systems strategising for universal access to healthcare.

### Social relations and accessing care

Extant literature indicates that communities struggling to access services such as medication and treatment tend to activate and nurture alternative strategies, such as drawing on their social connections in order to meet their needs [11, 12, 14, 15]. Traditionally, households and families usually provide informal care to their loved ones such as the elderly and those with disabilities. In this type of care the relationship exists regardless of the need for care. The focus in this article is on the care that individuals and their households access by virtue of being part of networks of social relations, outside of the immediate family, that are generously reciprocal. These social connections include social networks, degree of social inclusion and social support, which in this paper are broadly referred to as ‘social relations’.

Social relations represent one way in which populations with limited access to healthcare resolve their social, economic and financial problems. Through these social relations, individuals and households are able to access a pool of resources embedded in the community when the need arises, and in ways that match their needs, protecting them from a downward socio-economic spiral. The support provided may include borrowing or lending a bicycle to transport the ill person, nursing care for the sick, food, accommodation, etc. Due to the unpredictability of future need, these exchanges are mostly indirect, meaning that the support is based on need and not necessarily on what the recipient has previously contributed. For example, an individual who receives care when they are ill may repay this social debt with food to someone in need, rather than the person who provided the care. This type of exchange is known as a generalised reciprocity [16], which is based on the assumption that any good turn will be repaid at some time in the future. These social relations are also characterised by a trust that individuals will comply with group expectations, in order to maintain their standing in the community [17].

Studies in the early 1990s noted that family networks were not effective safety nets for people affected by HIV at that time, because of the poverty of household members and kin [18]. However, in a more recent project within which the present analysis was performed, it was found that many households registered with chronic illness and death were not in severe economic hardship, a finding attributed to their ability to draw resources from a broader social network [19]. Ugandan rural households have a history of supporting individuals during crises [20]. Individuals experiencing chronic illness tend to move from the urban to their rural homes where they have social networks and resources. During the outbreak of the HIV epidemic in the early 1980s, there had been concerns among health policy-makers in Uganda that there might be catastrophic socio-economic outcomes as individuals in need of care over-stretched resources within their social networks [21]. However, social relations proved to be flexible and adaptive in the face of these trials and with the arrival of antiretroviral (ARV) drugs, in tandem with establishment of community-based care groups, the severe threat of social collapse was averted.

### Access to health services in rural Uganda

The rural districts in Uganda are served by health centres that range from level I (lowest —village level) to level IV (highest—district level) and generally focus on outpatient services. The point of access to the health system is the level I health centre, which refers complicated cases to a health centre at the next level. However, the public health facilities are few in the rural areas, and are characterised by: lack of transportation for emergency cases from home or with referral between facilities, constant shortage of medication and chronic understaffing by health workers, with a ratio of one health worker to more than 20,000 people [22]. Therefore, more than 50% of the rural population depend heavily on private providers, spending on consultation and medicines, and incurring between 23 and 38% catastrophic expenditures [23].

The Ugandan government is seeking to provide universal access to healthcare services by applying strategies such as elimination of user fees in public health facilities, providing free treatment and introducing health insurance. However, in rural areas, where 84% of Ugandans live, non-medical needs such as transportation to health facilities, food and sustaining livelihoods while experiencing illness are still a burden for households [9, 24]. Providing free treatment and medication at national policy level has therefore not eliminated financial hardship for households in rural areas, while health insurance schemes have been unsuccessful partly due to unaffordability and lack of ownership among the rural community [25]. Such households may exploit other strategies that provide wider social protection, such as drawing on social relations to meet their needs during chronic illness and to limit potential financial hardships associated with ill health.

## Methods

### Study setting and sampling

This study was nested within the Rural Livelihoods Study (RLS) [19], which was itself based in a larger General Population Cohort (GPC) of the Medical Research Council/Uganda Virus Research Institute Research Unit on AIDS, a cohort of 20,000 people based in Kalungu district [26]. The cohort is open, allowing immigrants to the area and children born since establishment of the cohort to join. This cohort has been followed for over 25 years (since 1989) by researchers from the Medical Research Council (MRC). The GPC runs a clinic and a clinical laboratory at the field office, staffed at the time of this study by three doctors accessible on demand, and two full time clinical officers, and stocks most of the drugs on the essential drug list for Uganda. This quality of health facility is not common in the country, hence making the GPC clinic popular in this area. In-migration of people, mainly relatives to those already living in the area, in need of health care is common, and in these cases such people are included in the health system. The population served by the clinic lives within a five-kilometre radius, however due to poor road network access can be difficult. Travel is by bicycle or on foot, or at best by motorcycle or bicycle-taxi, as is the case in most rural Uganda. The MRC provides transportation for those individuals that are enrolled into the rural clinical cohort (RCC) for HIV care (Pre-ART and ART) for easy follow-up [27]. Even though health services are better here than in most rural areas, the findings will show that people still struggle to access services and to cope with meeting other needs when becoming ill. In areas with worse access to health services, struggles are likely to be harder, and community reciprocity is likely to be even stronger than in the cases presented here.

People live in semi-permanent structures built from locally available materials. The community consists mostly of people from the Baganda tribe, with 15% being of Rwandese origin, who are well assimilated. Religious affiliation is mostly Christian, with a significant Muslim minority (28%). Levels of literacy are low and the main income-earning activities are growing bananas, coffee and beans, and trading fish. In 2013, the prevalence of diabetes in this community was 0.4%, hyperglycaemia 2.9%, high blood pressure 22.5% and HIV 7.3% [4].

The survey data from the GPC households were checked for death of an HIV-positive adult over the previous 20-year period and divided into two groups; those with and those without an AIDS-related death. From each of the categories, a random sample of 200 households was selected for the RLS. The first 100 households from each category were traced for interviews, while the other 100 households were used for replacing dissolved/emigrating and unwilling households. Social and economic data, on e.g. asset ownership, cropping patterns, individual relations and income sources, were collected from households and used to examine how the epidemic had affected progress and livelihoods.

For the present study, a sub-sample of 22 households was drawn from the RLS for qualitative, in-depth analysis of households’ ways of supporting themselves in the event of illness or death, Table 1. These households were purposively selected to represent equal numbers of female- and male-headed households. In addition, the households were chosen to represent equal numbers of those affected by HIV and non-affected households. The purpose of these selection criteria was to assess whether gender of household head or HIV status affected households’ way of meeting their needs during chronic illness. However, when in-depth data were collected, it emerged that considerably more than half of the households selected, 19 out of 22 (86%), had experienced long-term illness or death of an adult household member, due to HIV infection and other causes.

**Table 1:** Age, sex and HIV status of head of households in the qualitative study

Household characteristics	Gender of head of household	
	Male	Female
Not affected by HIV	4	5
HIV-affected	6	7
Age group of head of household (years)		
≤ 45	1	3
> 45 to ≤ 60	4	5
> 60	5	4

### Data collection

The life history approach of obtaining information on the subjective essence of the entire life of respondents was used, because it is more holistic and provides in-depth knowledge about individuals’ daily life and various aspects of adaptive responses to changes and opportunities [28]. Data were obtained about the time of establishment of the household, status of assets owned over time and changes in membership, size and structure. The account of the individuals’ lives included their movement into and out of the household, births, illnesses, death, education and work events. Two local research assistants, one male and one female, carried out interviews in households and recorded changes in livelihood sources, household composition and structure. This was done during monthly two-hour visits to each household over the course of a year (2009/2010). The time was spent with the household head, spouse or any other adult household member, walking around compounds, fields and work premises, participating in activities and asking about on-going activities and recent and past events. The combination of life histories, serial interviews and observations made it possible to validate the data, thus increasing the consistency of data and the reliability of the findings [29]. The research assistants made notes of their visits, interviews and conversations, from which they produced detailed accounts of what was said and what they saw. The field notes were written in English and sometimes in Luganda, which is the most common local language. Additional observation visits were carried out to some households and around the study area. The research team gathered additional data on issues that, although external to the households, nevertheless affected them, such as prices, seasonality, crop changes, drought and political events.

### Data analysis

Data analysis was performed in three phases. In the first phase, interview summaries were analysed after each household visit, paying attention to emerging themes [30–32], to identify areas for further exploration and comparison. Conducting interim analysis alongside data collection shaped and gave direction to the inquiry for each household during the study. Any patterns identified were explored across households, while deviating stories (households that had unique experiences) that were important for the study were examined in-depth. The field summaries from the two research assistants were compared to ensure that they dealt with key issues, such as who should be defined as a household member, in the same way.

Coding was done manually and from the coding list categories were derived inductively [33]. From the data, primary patterns were clustered and then regrouped into sub-categories, followed by categories and finally broad themes. For example, the broad theme ‘Household experience of chronic illness’ covered: households that had a member with chronic illness, households which took in an ill person to provide care in exchange for other resources and households providing care for another household with an ill member in exchange for other resources. The broad theme ‘Responding to needs during illness of adults’ included categories such as: accessing free treatment and medication, helping a neighbour or friend to access care, moving to another household to receive care, relocating to provide care for an ill person in another household and exchanging care support with a neighbour.

By the end of the 12 visits to the households, some themes common to all households were identified. One in particular was that households drew support from friends, relatives and neighbours to meet their needs, including during illness. We explored this theme in depth in a second phase of analysis.

The second phase of the analysis focused on the households that reported providing care to ill individuals at least once in the 20 years preceding 2010. The data obtained were analysed to determine household experience of accessing free treatment and mechanisms by which households drew support to address needs during illness.

In the third phase of analysis, data from the histories of households that had experienced providing care to ill individuals were assessed and important processes leading to accessing care identified. In this phase, all available information on these households, including relocation of children, weather events and many other issues, was analysed. The objective was to determine whether access to healthcare and support was related to ability to draw support from social relations and how these social relations were sustained.

## Results

### Summary of household characteristics and experience of chronic illness

All households that participated in this study were located in an area with access to free healthcare services from the MRC since 1989. In Table 2 we present experiences of and responses to chronic illness in the households from Table 1. Here we can see how common some of these experiences and responses were.

**Table 2:** Experience with chronic illness reported by the household members in the qualitative study

	Sex of head of household		HIV status of household	
Household experience of chronic illness	Male	Female	HIV-non- affected	HIV-affected
Had a member with chronic illness	9	7	3	13
Provided care to a household with an ill member so as to access other support	0	3	3	0
No experience of chronic illness	2	1	3	0
Response to needs during chronic illness of adults				
Accessed free treatment and medication	10	12	9	13
Exchanged support with a neighbour and friend to access care	5	4	2	7
Ill person moved to another household for care	1	2	1	2
Relocated to care for ill person in order to access other support from their household	0	3	3	0

Nineteen of the 22 households studied reported providing care to chronically ill individuals (who were not necessarily HIV positive) at least once in the 20 years preceding 2010. This experience with illness was either direct, involving a household member (16/19, i.e. 84%), or indirect by having to provide support for a household with an ill person, in exchange for support to meet their own needs (16%). Of the 16 households reporting experience of an ill household member, nine had provided support to neighbours and friends with other needs (by taking in children, providing food, share-cropping, etc.) in exchange for care (bicycle transport to hospital, bedside care in hospital, help with household chores, gathering food and fuelwood from the fields). A further three households reported that their ill member moved to close relatives who were able to care for them when they were ill. In some instances, households reported providing care at one point in time and receiving care at another or exchanging support at one time and relocating to another household for care at another time.

### Experience with meeting needs during illness of household members

In this study, households that had a chronically ill member sought to address the need for healthcare in various ways. These included: 1) Free treatment and medicines; 2) depending on neighbours and friends to communicate with the clinic and to provide care; 3) building on and maintaining long-term social relations to access care; 4) taking in individuals with other needs in return for support with care; 5) re-grouping ill and healthy individuals so as to exchange support and care; and 6) continuously identifying new relationships through which to obtain resources. In this way, household members minimised the risk of exposure to financial hardships during chronic illness. We select examples from four households to illustrate the various experiences of chronic illness and accessing care. These were: a female-headed, HIV-affected household (Patricia’s story), a male-headed HIV-affected household (Kalooli’s story), and two female-headed HIV-non-affected households (Bettina’s and Eseri’s stories). We use the stratification applied in the RLS of HIV-status of the household and gender of the head of household.

### Accessing free treatment and medication

Free treatment and medication was available at the MRC health facility for all households in the study area. For services not offered at the facility, individuals received referrals to other health centres where they could receive appropriate healthcare, with the costs covered by the MRC. However, while treatment and medication were free to all, individuals who were part of the RCC and had enrolled for antiretroviral therapy (ART) received additional support, such as transportation. This eliminated most of the potential barriers to accessing care, such as unavailability of treatment and medication and high cost of treatment and transportation. Patricia’s story illustrates how chronically ill individuals surveyed in this study accessed healthcare and the kinds of resources they needed.

*At the time of interview, Patricia, in her 60s, rented a room at the trading centre for use as both business premises and living quarters, having returned 10 years previously from another town where she had gone to find work in the 1980s. She had moved back due to ill health and in order to access the free healthcare services that were now available in her village. When she was not ill, she sold alcohol and fresh foodstuffs for income to pay her rent, buy food and other necessities and hire labour for cultivating crops on her land, located in another village.*

*Where would I have got money for treatment? The only answer for me was death. But now I receive treatment for my disease and my life is better. I am thankful to the MRC* [clapping her hands] *because they pay all my bills when I am hospitalised …*

*Patricia started to access free basic treatment and medicines from the MRC clinic in 2000 and in 2004, when the MRC started providing ARV treatment to eligible clients, she was one of the beneficiaries.*

In addition to free ARVs and hospital care, Patricia was provided with transport to the health facility for follow-up visits and sometimes when she needed a medical consultation. With this available free treatment and transport, Patricia accessed adequate healthcare and did not fall into financial hardship. While free treatment and medication were available to everyone in the study area, free transport was only available to those on ART, and households with chronically ill members also had other needs such as food and help with personal care. One way of meeting these needs was through drawing support from their friends, neighbours and relatives.

### Depending on neighbours and friends to access treatment and care

In addition to the need for treatment, individuals also needed to communicate with the health facility and needed transportation (for those not on ART), food and care while they were ill, which were usually not affordable to individuals. To meet such non-medical needs, household members mostly drew support from their social relations in various ways. In Patricia’s household, in addition to treatment and transportation, she needed to communicate with the health facility and did not have access to a phone/reception. She reported:

*When I am sick my neighbour goes to the MRC clinic to report about my sickness and a clinic van is sent to pick me up…*

It was also common for individuals such as Patricia to get such support from neighbours, friends and relatives with food and care, as we can see if we continue to look at her story (which also reveals the problems of being absent from farming).

*However, due to the general food shortage in the community and her physical absence from her land, people in that village stole Patricia’s coffee and banana crop. She stopped hiring workers, cultivation on her land stopped and therefore she had insufficient income to buy food. When we inquired about her major source of food, she said:*

*My neighbour who sells meat gave me one kg of meat for free … My life is deteriorating. I don’t see why I should be bothered (to grow food) when all I need is a little food for me.*

*In addition, Patricia had an ‘auntie’* [her late mother’s best friend, whom Patricia now refers to as her aunt] *who frequently visited her and helped to sell alcohol and do other house chores such as cooking food and helping with personal hygiene, as well as bedside care when Patricia was hospitalised. In return for the help, this ‘auntie’ grew her crops on Patricia’s land in the other village.*

Thus through her social relations, Patricia benefited from the wider social protection she needed in order to meet other basic needs in her situation, including communication with the health facility, care and food. This support from neighbours and ‘family’ minimised the financial risks in accessing care. Patricia was in a position to repay the support her ‘auntie’ provided by allowing her to grow crops during the planting seasons. Through this exchange of services, Patricia was able to maintain a relationship that supported her during periods of illness.

### Building on and maintaining long-term relations to access care

It was common for households experiencing chronic illness to cite friends, neighbours and relatives as major sources of support. Some households, such as that of Kalooli described below, believed that they needed to build and maintain good relationships with relatives and friends in order to meet their future needs.

*Kalooli, in his 80s at the time of interview, lived with his teenage grandson in a three-room house. He owned about 1.5 acres of land, most of which had been cultivated by a neighbour for the previous two years on a crop harvest-sharing basis, whereby Kalooli received part of the crop. During the 1990s, Kalooli sold part of his land to support his household when his wife, son and daughter became ill and died. During the interviews, Kalooli was ill on a number of occasions and was therefore unable to work and provide for the household. His grandson did casual work for neighbours for food and money. Kalooli was not entitled to transportation from the clinic and he claimed that he could not receive hospital care as he had no one to provide bedside care in hospital and supply food (which is not included). He described his access to care during one of the visits when he was ill:*

*If I had someone to take care of me there I would have gotten hospitalised. They* [the hospital management] *cannot admit you when you have no one to care for you, cook and provide food.*

*Kalooli depended on his neighbours to transport him to the health centre and to provide care, including food, help with finding other treatment and personal hygiene.*

*When I was ill and unconscious my neighbours called a health worker from the neighbourhood to treat me.*

Providing and receiving generalised reciprocal support maintained connections with the social network. Households such as Kalooli’s regarded being able to give and receive support as an ‘investment’ with benefits that could be reaped when need arose in the future.

*Helping my neighbour is like giving a loan, which will be repaid when I am in need. If you do not give* [support] *what will you do when it* [crisis] *happens to you? In the past when I trapped mudfish I gave my neighbour some fish for free, even when they wanted to pay, and now those neighbours meet all my needs when I am ill…. I have debts that are not yet paid because I have been ill; I have to give bark-cloth* [produced from Natal fig, locally known as “mutuba” tree, and given as presents on important occasions such as death and marriage] *to the home where a man died in this village; and I have not yet given a bunch of matooke* [plantain, which is a staple crop] *to my neighbour whose son was recently married.*

Households in the study area thus endeavoured to maintain connections with their social network in order to access resources embedded in the social relations when they are in need. During times of illness, Kalooli’s household depended heavily on support from neighbours. While hospitalisation costs would have been covered by the MRC, Kalooli could not access this service as he had no-one to accompany him to hospital and provide care and food. His case revealed the dynamics involved in maintaining social connections, including giving support to others when they need it. This enabled households in the study area to meet their needs, especially during crisis such as chronic illness, confirming the critical role of social relations in providing the wider social protection required by households with limited financial resources. The stories told by Patricia and Kalooli were common among the study respondents and illustrate the importance of having free treatment and medicines but also of having support with non-medical needs during chronic illness.

### Individuals relocating so as to exchange care for other support

Some households that did not have a member with chronic illness, but had other needs, offered care services to households that were experiencing chronic illness and had resources, in exchange for other support. Unlike Patricia, Bettina had not experienced illness directly in her household initially.

*Bettina, who was in her 60s at the time of interview, lived with her older sister, her niece Peace and Peace’s four children. The household had 7.5 acres of land where they grew crops such as coffee and bananas and reared cows and goats for sale and subsistence. They had all moved to live in the household, now headed by Bettina, at different times so as to meet their needs, including a need for care.*

*Bettina had relocated to the study area in 1987 to help her parents, who were chronically ill and lived on their own. They needed treatment, food, care, personal help with household chores, transportation to medical facilities and work in the fields to produce food, among other things. At that time, she was 45 years old, childless and experiencing partner violence in her marriage, all of which prompted her to move back to her village. At her parents’ home, Bettina had accommodation and a field to cultivate crops for subsistence and income, and helped her parents in other various ways.*

*Bettina’s mother died in 1991 following a snake bite. Her father fell off his bicycle in 1990 and suffered a spinal injury and five years later he died. Therefore, in 1995 the status of the household changed from being male-headed to female-headed, with Bettina as the head. During his illness, Bettina took her father to hospital on a bicycle, washed him and provided food. She also paid a local herbalist to provide treatment using herbal concoctions, and personal care like washing the patient. She sold her coffee stocks so as to meet the bills for care.*

By moving to stay with her parents, Bettina provided the support they needed during chronic illness, including accessing treatment and medicines, food, transportation and personal care. This protected them from exposure to financial risks from e.g. having to sell assets to address their needs. At the same time, she met her own need for accommodation and food.

### Individuals with chronic illness re-locating to households with resources

In some households experiencing chronic illness, the individual who was chronically ill moved to live with relatives (or non-relatives) that had resources and were therefore in position to provide support. Bettina’s household (as the story unfolds further) also illustrated a situation where individuals who were chronically ill moved into the household to access support.

*With food, coffee bushes and income from crop sales, Bettina had the resources to support not only her parents, but also other relatives with various needs. In 1993, her brother, who lived in the fishing islands in Lake Victoria, moved to the household because he had developed HIV-related complications and needed to access the free treatment from the MRC and care from Bettina. Bettina transported him to the MRC clinic to receive treatment and medicine. Her brother also continued commuting to the lake and spent most of the day fishing, not only to generate income for the household, but also to keep away from being seen by the people in his community since he had developed rashes on his skin, a symptom the local people associated with HIV infection. Bettina commented:*

*The MRC counsellors advised him to stop going to the lake but he would not. He had developed rashes all over his body and did not want to be seen by people, he also needed money for hiring people to plant potatoes.*

*For one year this brother was bedridden and Bettina took care of him until he died in 1994. When asked about his contribution to the household Bettina responded:*

*He was the one planting potatoes when he was not sick, he brought fish which we sold and part of it was for home consumption . . . however his body did not respond to the treatment from MRC because he consumed alcohol. He was therefore too weak; he could not wash our father who was bedridden. Imagine, I am a woman but I had to care for him and also wash my father…*

*An older sister to Bettina, who lived alone in another village, also joined the household. She had chronic illnesses including back pain and visual impairment, and needed to access the free treatment and other support including transportation to the health facility, care and food.*

*In 2003 Bettina herself developed chronic illness and therefore needed healthcare services. Both Bettina and her sister accessed the free medicines available at the MRC. However, the household needed other help to provide e.g. food, care and transportation. In order to meet these needs, their teenage niece called Peace (aged 17 years) joined the household to provide care for Bettina and her sister. Peace had left school due to lack of money to pay the fees and needed support for accommodation and food. She carried out most of the work in the fields and household chores. She also organised transportation to the MRC clinic and provided personal care and food. At the same time, she met her needs for accommodation and food. During the following three years, Peace had two sets of twins with a man who lived in another village and did not provide any support for the children. Bettina and her sister provided childcare and lodging for Peace’s children in exchange for the care that they received.*

*Bettina and her sister had ailments that were not addressed within the available basic health services at the MRC. Their brother who lived in the city took them in turns to the city to get treatment. During one of the visits, Bettina’s sister commented:*

*People can die before their time due to lack of treatment. People in this village said that I was suffering from old age. At the city hospital I received blood, urine and stool tests and they gave me medicines. Now I feel better.*

In summary, since Bettina’s household had accumulated resources, including food, income, accommodation and manpower, it was able to support several individuals who had health related and non-health related needs such as care, food, accommodation, child care and transportation. All these were gathered under one roof and thus the household was at one point in time supporting others and at another point in time receiving support. Accounts such as Bettina’s were common among the study households.

### Expanding social relations for additional resources

Continuously expanding the number of people with whom to exchange support was an important form of ‘insurance’ for households experiencing chronic illness and for households that had other needs. The continuation of Bettina’s story illustrates that.

*Over time, as Bettina and her sister needed more care, Peace could not provide the care and do all the field work by herself, as well as take care of her children. Therefore Bettina identified a single mother from the neighbourhood who had no land to cultivate and who moved around looking for work in exchange for food. This woman was recruited to help Bettina’s household with work in the fields and household chores. The woman did not always get paid immediately for her work, but Bettina provided support to her whenever she needed it. Bettina explained:*

*There is a neighbour who works in the fields for free. She is my friend. She stays in this village but she does not own land so she goes around looking for food and money. I provide her with cassava and potatoes and other necessities like soap and salt even on occasions when she has not worked for me. I sometimes hire her to hoe (weed) through the cassava, potatoes and banana plantation. When the harvest is ready she gets to harvest the crop in return for her labour.*

*Bettina’s household also regularly provided food for another neighbour who was ill and lived on her own. This neighbour gave nothing to Bettina in return because she was not able. When asked about giving out support Bettina responded:*

*There is an elderly woman that I always give food. She is sickly and weak and lives on her own. All her children live in the city and rarely visit her… I do not get anything from her in exchange for the food.*

*Bettina thought that it was good will to support others in the community:*

*In this community you will always get something for free when need arises, such as seeds when the planting season is here, food when the harvest is ready …*

The food, housing and land which Bettina’s household possessed placed it in a position to create new relations and exchange care for support, and also to provide support to those who needed it without receiving anything in return.

In the same way, households that did not have a chronically ill member but had other needs, such as food and accommodation, were able to turn to relatives and create new friends with whom to exchange support.

*Eseri was in her early 30s at the time of interview and, like Bettina, she had moved into the study area to live with and care for her elderly grandmother, along with her four children. Her grandmother was chronically ill and lived alone on 11.0 acres of land with coffee and matooke, while Eseri needed food and housing for her household. Like Bettina, Eseri provided care for her grandmother, including personal care and food, took her to hospital and also tended the plantation. In exchange, she had housing and food for her family. Following the death of her grandmother two years later, she inherited the house and two acres of the land. Most of the land was rocky and therefore not suitable for growing crops. Unlike Bettina’s household, which had land to cultivate, Eseri needed to find other ways to support her household with food and money. She identified a couple in the neighbourhood who were chronically ill and helped them cultivate and pick coffee. The couple provided Eseri food and money in return, and even on some occasions when Eseri had not helped them. A year later the man died, leaving a widow in her 60s. Eseri had this to say during one of the interview visits:*

*The bad news here is that my neighbour died. He was very kind, he always gave me salt, cooking oil, matooke for free … His wife is not as kind, but she now lives on her own. She is old, sickly and weak. I always prepare food, fetch water and wash clothes for her. When I cook we (Eseri and her household) all eat from there. When I work on her farm I receive matooke for my family or a bucket of coffee, which I sell and buy food. Her sons live in the city and visit once in a while…*

*Eseri spent at least four days a week in the other household, providing personal care, food, taking the widow to hospital and tending the farm. In return, she received the food that her household needed and also money for her other needs. Eseri’s household was also able to receive support in advance that she paid back when she was able:*

*This month my family and I had malaria and diarrhoea. I was too weak to do any work and the children were too weak to go to school. The widow that I always help provided the money, 20,000 UGX [equivalent to six USD] which I used to buy medicines and food from the shop. The youngest did not respond to the medicines, I had to use the rest of the money to take him to the MRC clinic. We are now digging in the widow’s coffee plantation to repay the debt.*

Thus Eseri had helped two households with individuals suffering chronic illnesses. In the first case she relocated her family to her grandmother’s house. She therefore accessed accommodation and food and later inherited and owned property. Later when her grandmother died, Eseri helped a neighbouring household experiencing chronic illness, which enabled her to continue accessing food and money. In essence, through continuously expanding her social relations, Eseri was able to support her household. At the same time, she provided the wider social protection that her grandmother and neighbour needed during chronic illness, including accessing free treatment and medicines.

Bettina and Eseri’s households demonstrate the importance of continuously creating reciprocal social relations to meet increasing and changing household needs and also show that these social relations are not static, e.g. not having close relatives around did not mean that a household was vulnerable. The new relations sometimes transcended the close kin network and made it possible for households to meet their needs. It is also clear from their stories that everyone is expected to support people in need if they can, but that a basic level of trust in each other is a precondition for this support.

## Discussion

This study demonstrated the importance of social relations for a health system that is endeavouring to provide universal access to healthcare for low-income rural populations. The data from the households surveyed indicate that the free treatment and medicines provided at the health facility were very important for individuals experiencing prolonged illness. However, such individuals also needed broader social protection in order to meet other needs during illness and to prevent costs becoming catastrophic for the household. The households had developed an informal type of social protection composed of a network of kin, friends and neighbours.

It is obviously important for a health system to target specific components of healthcare such as access to treatment and medicines. However, any health system that is aiming at achieving broader outcomes, such as universal access to healthcare, needs to go beyond conventional healthcare practices and include a wider set of social and economic interventions. This will insure the population against risks and future insecurities. Recognising and supporting community health actors as part of a national health system is gaining ground due to their connections with others in the community and their non-bureaucratic elements composed mainly of trust, reciprocity and sustainability [34].

A previous study on five sub-Saharan African countries found that ‘hidden’ actors played a key role in access to care and hence contributed to improved child survival [35]. The examples provided in this paper, of the dynamics of social relations in rural communities, provide an indication why the spread of HIV has not led to the far-ranging socio-economic outcomes that were earlier feared, including eroding social networks [36, 37]. Due to the multiplicity of other wider impacts on communities, such as declining land holdings and droughts, individuals and households are apparently compelled to exchange support in order to meet their needs. Therefore, people needing extensive care usually provide support to those with other needs, thus engaging in a two-way exchange that strengthens rather than erodes social networks. This shows how social relations are constantly developing and adapting and are being re-negotiated to fit the current needs of households.

The unforeseen situations that increasingly typify the day-to-day lives of individuals in poor rural households, such as food shortages, prolonged illness, prolonged droughts and sudden death, force households in the area to develop an informal insurance system built on trust and general reciprocal exchange of support. This support based primarily on need extends beyond kinship and beyond direct exchange of services. Through trust and participation in reciprocal activities, individuals remain connected and cooperate for long-term mutual benefit. Such reciprocal activities in the study context of rural Uganda include care for the sick and frail, labour and food exchange, midwifery, child care, money-lending, funerals and weddings.

Furthermore, it is notable that the strong reciprocal social relationships that emerged in this study took place in the context of free healthcare from MRC, which could have been expected to solve most healthcare needs and lower the need for having to rely on kin, neighbours and the general community. It shows that availing the free healthcare addressed only one aspect of what households with sick individuals needed in this setting. It is also likely that the free healthcare allowed household members’ to concentrate time and resources in other areas of need. Thus, the provision of free health care could have had the additive effect that people were able to care for each other’s non-medical needs more.

A comprehensive social protection model that provides health services so that access by rural households is improved and that provides financial protection might be too resource-intensive for a setting with limited resources. Moreover, given that experiences with chronic illness and the types of needs can be context-specific, strategies and responses that are locally developed and embedded in that specific context are important to consider [38]. In the study area, problems with transport, long dry spells affecting food availability and out-migration of young adults exacerbate the challenges with accessing care during chronic illness. The individuals in the study area use their social relations to meet their needs, and this resource can be better integrated into discussions on improving health service provision for such areas.

In this analysis we found that involvement of neighbours and fostering of children in need had become increasingly important. The involvement of neighbours may have been driven by the increasing fragmentation of families due to out-migration, hence the neighbours becoming more important due to their proximity. Their participation in reciprocal exchanges of support increased households’ social assets. Drawing on neighbours was common when the needs within the close kin exceeded the available resources. Studies of orphan welfare in rural Uganda [39, 40] found that child fostering ensured the health and welfare of orphans, but that the children fostered were also an invaluable resource for the receiving household. It was also common for children in the households surveyed in the present study to participate in reciprocal activities in order to ensure continuous connection to the social network, from which the household could draw resources for current and future benefit.

The life history accounts in the present study indicate that there are several benefits from reciprocal social relations. One of them is being able to trust and expect that needs would be met by others, which gave individuals the motivation to invest in generalised reciprocal support, for example when Bettina commented on helping the elderly lady who could not help her back: “*In this community you will always get something for free when need arises”*. Individuals gave support without expecting immediate reciprocation, or even any reciprocation, from the recipient. In the same way, they were not always able to redeem support directly from the households and individuals they had previously helped when they needed support themselves. Their good will to give had been registered and was regarded as a good reputation by others in the community, and would most likely lead to being helped when in need. This way of investing for future eventualities is essential in relation to health care, especially due to variations in timing of individual health needs, such as during prolonged illness, and when the person in need can no longer contribute something in exchange for the care. Most importantly, this also illustrates a cohesive attribute of social relations that makes them a potential resource for a health system that is aiming to improve access to healthcare for its population.

Thus generalised reciprocal relations and trust provide a form of social protection to household members during prolonged illness, and hence financial protection. However, the individual and household material deprivations due to overall social and economic factors and the national political choices should not be downplayed. Such deprivations can influence the ability to participate in reciprocal activities and hence undermine access to health services and protection from catastrophic expenditure on health services. Moreover, social networks can be socio-economically costly, and these costs are borne more heavily by those with fewer economic resources and marginalised groups, such as women, children and the elderly. In this study, lack of resources such as land, food and accommodation was common among the women that relocated or cared for neighbours and friends. Social relations have also been associated with negative health outcomes such as stress, especially in situations where individuals with limited resources find it a burden to contribute to the network. Therefore, care should be taken to assess the quality of social relations and strengthen the aspects that are beneficial for healthcare strategies, while finding approaches to minimise those aspects that are harmful to health.

## Conclusions

This analysis of the experience of individuals in accessing free medication and treatment, their needs and the way in which they meet these needs during chronic illness provides unique insights for health systems seeking to improve access in rural communities in similar contexts. It was found that improving access to healthcare while providing financial protection for the rural poor depends on broader social protection. In the rural population studied, this broader social protection was achieved through reciprocal exchange of support to address healthcare needs, hence mediating access to free medical care and also meeting other needs.

## Abbreviations

ART: Antiretroviral
GPC: General Population Cohort
HIV: Human Immunodeficiency Virus
MRC: Medical Research Council
NCDs: Non-communicable Diseases
RCC: Rural Clinical Cohort
RLS: Rural Livelihoods Study

## References

[1] Nixon SA, H-H J, Whiteside A, Barnett T (2011). The increasing chronicity of HIV in sub-Saharan Africa: re-thinking “HIV as a long-wave event” in the era of widespread access to ART. Glob Health.

[2] World Health Organization (2002). Innovative care for chronic conditions: building blocks for action.

[3] World Health Organization (2014). Global status report on noncommunicable diseases 2014.

[4] Maher D, Waswa L, Baisley K, Karabarinde A, Unwin N (2011). Epidemiology of hypertension in low-income countries: a cross-sectional population-based survey in rural Uganda. J Hypertens.

[5] Kipp W, Kamugisha J, Jacobs P, Burnham G, Rubaale T (2001). User fees, health staff incentives, and service utilization in Kabarole District, Uganda. Bull World Health Organ.

[6] Barennes H, Frichittavong A, Gripenberg M, Koffi P (2015). Evidence of high out of pocket spending for HIV care leading to catastrophic expenditure for affected patients in Lao People's Democratic Republic. PLoS One.

[7] World Health Organization (2005). The elimination of user fees in Uganda: impact on utilization and catastrophic health expenditures.

[8] Orem JN, Mugisha F, Kirunga C, Macq J, Criel B (2011). Abolition of user fees: the Uganda paradox. Health Policy Plan.

[9] Kansiime C, Rutebemberwa E, Mugisha A, Mugisha S, Asiimwe BB, Rwego IB (2014). Determinants of Patients' choice of provider in accessing brucellosis care among pastoral communities adjacent to Lake Mburo National Park in Kiruhura District, Uganda. PLoS One.

[10] Ayé M, Champagne F, Contandriopoulos A-P (2002). Economic role of solidarity and social capital in accessing modern health care services in the Ivory Coast. Soc Sci Med.

[11] Manderson L, Block E, Mkhwanazi N (2016). Fragility, fluidity, and resilience: caregiving configurations three decades into AIDS. AIDS Care.

[12] Manderson L, Block E (2016). Relatedness and care in southern Africa and beyond. Soc Dyn.

[13] Thoits PA (2011). Mechanisms linking social ties and support to physical and mental health. J Health Soc Behav.

[14] Kawachi I, Kennedy BP, Glass R (1999). Social capital and self-rated health: a contextual analysis. Am J Public Health.

[15] Putnam RD (2000). Bowling alone: the collapse and revival of American community: Simon and Schuster.

[16] Sahlins MD (1972). Stone age economics.

[17] Hart K. Kinship, contract, and trust: the economic organization of migrants in an African city slum. Trust: Making and breaking cooperative relations. 2000:176–93.

[18] Seeley J, Kajura E, Bachengana C, Okongo M, Wagner U, Mulder D (1993). The extended family and support for people with AIDS in a rural population in south west Uganda: a safety net with holes?. AIDS Care.

[19] Ekoru K, Bukenya D, Lutalo T, Pain A, Seeley J (2010). The longitudinal impact of HIV and AIDS on rural livelihoods in East Africa,(MRC/UVRI and RHSP Uganda component). A report for the food and agriculture organisation of the United Nations.

[20] Ntozi JP, Nakayiwa S, Orubuloye IO, John CC, James PMN (1999). AIDS in Uganda: how has the household coped with the epidemic. The continuing HIV/AIDS epidemic in Africa: responses and coping strategies.

[21] Barnett T, Blaikie P (1992). AIDS in Africa: its present and future impact.

[22] World Bank (2010). Fiscal space for health in Uganda.

[23] Kwesiga B, Zikusooka CM, Ataguba JE (2015). Assessing catastrophic and impoverishing effects of health care payments in Uganda. BMC Health Serv Res.

[24] Musoke D, Boynton P, Butler C, Musoke MB (2014). Health seeking behaviour and challenges in utilising health facilities in Wakiso district, Uganda. Afr Health Sci.

[25] Whiteford P (2008). How much redistribution do governments achieve? The role of cash transfers and household taxes. Growing unequal?.

[26] Asiki G, Murphy G, Nakiyingi-Miiro J, Seeley J, Nsubuga RN, Karabarinde A (2013). The general population cohort in rural south-western Uganda: a platform for communicable and non-communicable disease studies. Int J Epidemiol.

[27] Bukenya D, Wringe A, Moshabela M, Skovdal M, Ssekubugu R, Paparini S, et al. Where are we now? A multi country qualitative study on access to pre-ART care services, a precursor for ART initiation. Sex Transm Infect. 2017; 10.1136/sextrans-2016-052970 PMC5739845 28615327

[28] Ojermark A (2007). Presenting life histories: a literature review and annotated bibliography.

[29] Krause D (1989). The research act - a theoretical introduction to sociological methods, 3rd edition - Denzin,Nk. Teach Sociol.

[30] Mills M, Huberman M. Qualitative data analysis: An expanded sourcebook (2nd Edition). Thousand oaks: Sage Publications, Inc.; 1994.

[31] Merriam SB (2009). Qualitative research: A guide to design and implementation: 3rd ed.

[32] Lincoln YS, Guba EG (1985). Naturalistic inquiry.

[33] Pope C, Ziebland S, Mays N (2000). Qualitative research in health care: Analysing qualitative data. BMJ: British Medical Journal.

[34] Schneider H, Lehmann U (2016). From community health workers to community health systems: time to widen the horizon?. Health Systems & Reform.

[35] Leon N, Sanders D, Van Damme W, Besada D, Daviaud E, Oliphant NP, et al. The role of ‘hidden’ community volunteers in community-based health service delivery platforms: examples from sub-Saharan Africa. Glob Health Action. 2015;8 10.3402/gha.v8.27214. 10.3402/gha.v8.27214 PMC4359271 25770090

[36] Drinkwater M, McEwan M, Samuels F (2006). The effects of HIV/AIDS on agricultural production Systems in Zambia: a restudy 1993–2005. IFPRI RENEWAL report.

[37] Hosegood V (2009). The demographic impact of HIV and AIDS across the family and household life-cycle: implications for efforts to strengthen families in sub-Saharan Africa. AIDS Care-Psychological and Socio-Medical Aspects of AIDS/HIV.

[38] United Nations. Community Realities & Responses to HIV/AIDS in Sub-Saharan Africa. United Nations, New York: United Nations Office of the Special Adviser on Africa; 2003.

[39] Kasedde S, Doyle AM, Seeley JA, Ross DA (2014). They are not always a burden: older people and child fostering in Uganda during the HIV epidemic. Soc Sci Med.

[40] Rutakumwa R, Zalwango F, Richards E, Seeley J (2015). Exploring the care relationship between grandparents/older Carers and children infected with HIV in south-western Uganda: implications for Care for both the children and their older Carers. Int J Environ Res Public Health.

